# Interventions for Reducing Suicide Risk in Cancer Patients: A Literature Review

**DOI:** 10.5964/ejop.v15i3.1741

**Published:** 2019-09-27

**Authors:** Giedre Bulotiene, Kamile Pociute

**Affiliations:** a Department of Physical Medicine and Rehabilitation, National Cancer Institute, Vilnius, Lithuania; b Clinic of Psychiatry, Institute of Clinical Medicine, Faculty of Medicine, Vilnius University, Vilnius, Lithuania; Webster University Geneva, Switzerland

**Keywords:** cancer, suicide, prevention, interventions, risk factors, mental health

## Abstract

The suicide risk of people diagnosed with cancer is two times higher than the general population. The number of cases of diagnosed cancer is estimated to rise by 70% over the next two decades. Evidence-based prevention strategies are necessary to protect this vulnerable group of individuals. The purpose of this review was to find out the risk factors of suicide and which types of interventions can serve as prevention strategies. Psychosocial interventions, pharmacotherapy and physical activity can play a preventive role in reducing psychosocial and physical risk factors, such as mental disorders, poor social support, poor performance status and pain. Further research is needed to develop effective suicide prevention strategies for cancer patients.

Cancer is the second most common cause of death globally. The number of cancer cases is predicted to rise by 70% over the next two decades (WHO, 2017). The suicide rate of oncological patients is approximately two times higher than the general population (Misono, Weiss, Fann, Redman, & Yueh, 2008; Oberaigner, Sperner-Unterweger, Fiegl, Geiger-Gritsch, & Haring, 2014). When the disease reaches an advanced stage, the risk rates increase even further (Kaceniene, Krilaviciute, Kazlauskiene, Bulotiene, & Smailyte, 2017; Oberaigner et al., 2014). It has been found that among cancer patients, the increased suicide risk can persist for up to 15 years after the diagnosis (Misono et al., 2008). A study found that cancer is one of the very few physical diseases to actually increase the risk of suicide (Bolton, Walld, Chateau, Finlayson, & Sareen, 2015).

There are several known factors that increase the risk of suicide in oncological patients: older age at the time of diagnosis, a lower level of education, non-marital relationship status, a rural area of residence, psychological comorbidity, several specific types of cancer, advanced stages of cancer, atheism, and poor performance status (Hultcrantz et al., 2015; Oberaigner et al., 2014; Shim & Park, 2012; Smailyte et al., 2013). 41.7% of all cancer patients have psychiatric disorders (Gopalan, Karunakaran, Prabhakaran, & Jayakumar, 2016). The most common psychiatric disorder among cancer patients is depression (de la Grandmaison, Watier, Cavard, & Charlier, 2014). Depression in the oncological population is the leading risk factor for suicide (Diaz-Frutos, Baca-Garcia, Mahillo-Fernandez, Garcia-Foncillas, & Lopez-Castroman, 2016). Therefore, identifying depression and its symptoms is essential for assessing the suicide risk factors and choosing fitting interventions. Several psychological tests used for identifying depression are as follows: The Beck Hopelessness Scale; Diagnostic and Statistical Manual of Mental Disorders, Fifth Edition criteria; Patient Health Questionnaire; Endicott criteria using the Hamilton Depression Rating Scale; the question: "Do you feel depressed?" and others (Anguiano, Mayer, Piven, & Rosenstein, 2012; Hughes, 2016). 6–25.24% of cancer patients have suicidal thoughts (Diaz-Frutos et al., 2016; Leung et al., 2013; Zhong et al., 2017). It was found that 30% of patients with suicidal thoughts or intentions do not actually have depression (Leung et al., 2013). Several studies found that demoralization was a more significant factor in encouraging suicidal thoughts than depression (Fang et al., 2014; Vehling et al., 2017). It has also been found that 21% of cancer patients experience a clinically significant form of demoralization syndrome. 14% of cancer patients are only diagnosed with demoralization and no other psychological co-morbidities (Vehling et al., 2017). Demoralization in cancer patients can be evaluated by using the Demoralization Scale-II (Robinson, Kissane, Brooker, Hempton, et al., 2016; Robinson, Kissane, Brooker, Michael, et al., 2016).

There are several suicide prevention strategies based on the general population, such as psychological and pharmacological treatment of depression, restriction of lethal tools for suicide, and school-based awareness programs (Zalsman et al., 2016). However, there is a severe lack of suicide prevention strategies implemented for cancer patients, despite the fact that both the factors for increased suicide risk and the methods of assessing and preventing them are known. Therefore, in order to help reduce suicide rates of oncological patients, we have aimed this paper at understanding and creating adequate methods of suicide prevention by reviewing the known suicide risk factors and the already existing types of interventions implemented.

## Method

The databases of Pub Med and the Cochrane library were used to find all the relevant English language studies that were published between January 2007 and December 2017. The key words used were “suicide,” “cancer” and “prevention.” Of the 275 studies retrieved from the PubMed database, eight met the necessary criteria (Table 1), of which three studies were concentrated on interventions and five studies were focused on suicide risk factors (SRF) (Table 2).

**Table 1:** Eligibility Criteria

Criterion	Eligible values
Publication date	January 2007–December 2017
Language	English
Population	Oncology patients
Object	Suicide prevention (risk factors and/or possible interventions)
Study design	Randomized-controlled trial, systematic review, meta-analysis

**Table 2:** Selected Articles on Suicide Prevention Meeting the Eligibility Criteria

Study	Author, Year	SRF or Intervention
1. Suicide rates and risk factors among Korean cancer patients, 1993–2005	Ahn et al., 2010	SRF
2. Immediate risk of suicide and cardiovascular death after a prostate cancer diagnosis: Cohort study in the United States	Fang et al., 2010	SRF
3. Death by suicide and other externally caused injuries following a cancer diagnosis: The Japan Public Health Center-based Prospective Study	Yamauchi et al., 2014	SRF
4. Routine screening for suicidal intention in patients with cancer	Leung et al., 2013	SRF
5. Suicidality and its associated factors in cancer patients: Results of a multi-center study in Korea	Shim & Park, 2012	SRF
6. A randomized, placebo-controlled trial of citalopram for the prevention of major depression during treatment for head and neck cancer	Lydiatt et al., 2008	Intervention
7. A randomised controlled trial of a mindfulness intervention for men with advanced prostate cancer	Chambers et al., 2013	Intervention
8. Behavioral activation and problem-solving therapy for depressed breast cancer patients: Preliminary support for decreased suicidal ideation	Hopko et al., 2013	Intervention

*Note.* SRF = suicide risk factors.

Studies from The Cochrane Collaboration database were investigated, three studies met the necessary criteria, all of which were reiterated with articles in the PubMed. We assessed the suicide prevention strategies of the general population (Zalsman et al., 2016) and additionally researched the articles by using a variety of keyword combinations, such as: “cancer”, “suicide”, “prevention”, “education”, “social”, “networks”, “television”, “screening”, “screen”, “access”, “means”, “internet”, “depression”, “psychotherapy”, “antidepressants”, “physical”, “activity”. To improve the quality of our literature review, we decided to only include randomized-controlled trials, systematic reviews and meta-analyses in the “Interventions” section. A total of 53 articles were included in the literature review.

## Results

### Suicide Risk Factors

We divided the suicide risk factors (SRF) into two groups. We included sociodemographic and clinical SRF in the first group. The second group consisted of physical and psychosocial SRF (Table 3). Physical and psychosocial risk factors are especially important for suicide prevention, since they are more heavily influenced by interventions, as are the socio-demographic and clinical suicide risk factors.

**Table 3:** Suicide Risk Factors

1. SRF group	2. SRF group
1.1 Sociodemographic SRF Atheism (Shim & Park, 2012) White race (Misono et al., 2008) Male gender (Leung et al., 2013; Misono et al., 2008) Lower education (Ahn et al., 2010; Smailyte et al., 2013) Living in rural areas (Smailyte et al., 2013) Non-employment status (Ahn et al., 2010; Smailyte et al., 2013) Non-marital relationship status (Ahn et al., 2010; Fang et al., 2010; Misono et al., 2008; Smailyte et al., 2013) Older age at diagnosis (Kaceniene et al., 2017; Kendal & Kendal, 2012; Misono et al., 2008) 1.2 Clinical SRF First year after diagnosis (Johnson et al., 2012; Yamauchi et al., 2014 Specific types of cancer^a^ (Ahn et al., 2010; Kaceniene et al., 2017; Kendal & Kendal, 2012; Misono et al., 2008; Smailyte et al., 2013) Advanced cancer (Akechi et al., 2010; Fang et al., 2010; Kaceniene et al., 2017; Misono et al., 2008)	2.1 Physical SRF Pain (Park et al., 2016) Poor physical functioning (Robson et al., 2010; Shim & Park, 2012) 2.2 Psychosocial SRF Psychological comorbidity (Balci Sengul et al., 2014; Diaz-Frutos et al., 2016; Park et al., 2016; Shim & Park, 2012; Spencer et al., 2012): Depression Anxiety Panic disorder Posttraumatic stress disorder Poor social support (Balci Sengul et al., 2014)

*Note.* SRF = suicide risk factors.

^a^lung and bronchus, stomach, esophageal, pancreatic, colorectal, oral cavity and pharynx, larynx, head and neck, hematopoietic, genital organs, nasal cavity and sinuses, bone and articular cartilages.

### Interventions

Effects of interventions on SRF found in the literature are summarized in Table 4.

#### Psychotherapy, Psychosocial Interventions and Education

Meta-analysis showed that psychotherapy (supportive, cognitive, behavioral and problem-solving therapy) is effective in reducing depressive symptoms and psychological distress. Among incurable cancer patients, however, there was no evidence that psychotherapy was beneficial in improving the symptoms of anxiety (Akechi, Okuyama, Onishi, Morita, & Furukawa, 2008). Other research revealed the significant positive impact of music therapy and medicinal music interventions for anxiety, depression, pain, fatigue, and quality of life (QoL), and a decrease in heart and respiratory rate and blood pressure in adult and pediatric cancer patients. However, music intervention had no significant effect on mood, distress, oxygen saturation levels, spiritual well-being (adolescents or young adults) and physical functioning of people with cancer (Bradt, Dileo, Magill, & Teague, 2016). While supportive-expressive group therapy is associated with an improved rate of survival 1-year after the diagnosis and decreased pain scores in women with metastatic breast cancer, unfortunately no 5-year post-diagnosis survival benefits and no improvements on psychological measures were found after the therapy, according to Profile of Mood States test (POMS) (Mustafa, Carson-Stevens, Gillespie, & Edwards, 2013). Another study revealed that behavioral activation and problem-solving therapy for depression among breast cancer patients with major depressive disorder, are helpful in increasing hopefulness and reducing depression and suicidal ideation (Hopko et al., 2013). Psychosocial interventions (cognitive behavioral, psycho-educational, supportive, counselling) is helpful for improving the physical component of general health-related QoL, cancer-related QoL and the knowledge of prostate cancer of men with prostate cancer, but the findings for depression, distress, uncertainty, symptom-related QoL and self-efficacy did not achieve any statistical significance (Parahoo et al., 2013). A randomized controlled trial showed that tele-based group Mindfulness-Based Cognitive Therapy did not improve the quality of life and psychological outcomes of men with advanced prostate cancer when compared with those who received the minimally enhanced usual care (Chambers et al., 2013). Nursing interventions to manage breathlessness and nursing programs have a positive impact on the symptoms of depression, anxiety and other related types of distress, along with an improvement to performance status and the general satisfaction with care. Several studies have shown that telephone-based interventions for caregivers have beneficial effects as well. Telephone-based interventions for caretakers were effective at reducing signs of pain and depression, while improving physical well-being, quality of life and self-efficacy among cancer patients (Rueda, Sola, Pascual, & Subirana Casacuberta, 2011) (Table 4).

**Table 4:** Interventions for Reducing Suicide Risk Factors

Authors, year	Design	Population	*N*	Interventions	Effect of interventions	
					Positive effect	No effect
Pharmacotherapy						
Lydiatt et al., 2008	RCT	Patients with head and neck cancer	23	Citalopram hydrobromide	• Depression • Suicidality • Global mood state • QoL	
Lydiatt et al., 2013	RCT	Patients with head and neck cancer	148	Escitalopram	• Depression • QoL	
Ostuzzi et al., 2015	S/M	Cancer patients	861	Non-selective monoamine reuptake I; selective serotonin reuptake I; monoamine oxidase A I; non-selective monoamine oxidase I; newer and non-conventional antidepressive agents		• Depression
Psychotherapy, psychosocial interventions and education						
Akechi et al., 2008	S/M	Patients with advanced cancer	780	Supportive, cognitive behavioral and problem-solving therapy	• Depression • Psychological distress	• Anxiety
Bradt et al., 2016	S/M	Cancer patients	3,731	Music therapy and music medicine interventions	• Anxiety • Depression • Pain • Fatigue • QoL • Heart rate • Respiratory rate • Blood pressure	• Mood • Distress • Oxygen saturation level • Spiritual well-being • Physical functioning
Mustafa et al., 2013	S/M	Women with metastatic breast cancer	1,378	Supportive-expressive group therapy	• 1-year survival • Pain	• 5-year survival • POMS
Hopko et al., 2013	RCT	Cancer patients with major depressive disorder	80	Behavioral activation and problem-solving therapy	• Depression • Suicidal ideation • Hopefulness	
Parahoo et al., 2013	S/M	Men with prostate cancer	3,204	Cognitive behavioral, psycho-educational, supportive, counselling	• Physical component of general health-related QoL • Cancer-related QoL • Knowledge of prostate cancer	• Depression • Distress • Uncertainty • Symptom-related QoL • Self-efficacy
Chambers et al., 2013	RCT	Men with advanced prostate cancer	189	Group tele-based Mindfulness-Based Cognitive Therapy	• Mindfulness skill of observing	• Distress • QoL
Rueda et al., 2011	S	Patients with lung cancer	1,592	Nursing interventions to manage breathlessness	• Performance status • Emotional functioning • Symptoms • Panic episodes control • Depression • Anxiety	• Survival
				Structured nursing programs	• Symptom distress • Clinical deterioration • Dependency • Emotional functioning • Satisfaction with care	
				Counselling	• Depression • Life satisfaction • Self-esteem	
				Coaching sensory self-monitoring	• Information about pain	• Pain • Anxiety • Depression • Catastrophizing
				Telephone-based sessions for care-givers	• Pain • Physical well-being • Depression • QoL • Self-efficacy	
				Exercise	• Quadriceps strength • Self-empowerment	• QoL
				Nutritional interventions	• Energy intake	• QoL • Survival • Weight loss
				Reflexology	• Anxiety • Pain	
Exercises						
Levin et al., 2017	RCT	Cancer patients	32	Supervised exercises; self-managed home-based exercises	• Depression • QoL (mental health) • Body strength	• Anxiety • QoL (physical health)
Bergenthal et al., 2014	S/M	Patients with haematological malignancies	818	Aerobic physical exercise	• QoL • Physical functioning • Depression • Fatigue	• Mortality • Anxiety • Physical performance
Cramer et al., 2017	S/M	Women with breast cancer	2,166	Yoga	• Health-related QoL • Fatigue • Sleep disturbances • Depression • Anxiety	• Depression • Anxiety • Health-related QoL • Sleep disturbances
Furmaniak et al., 2016	S/M	Women with breast cancer	2,626	Aerobic or resistance or both exercises	• Physical fitness • Fatigue • Cancer site-specific QoL • Cognitive function	• Health-related QoL • Cancer-specific QoL • Depression
Bradt et al., 2015	S/M	Cancer patients	207	Dance/movement therapy	• QoL • Somatization • Vigor	• Depression • Stress • Anxiety • Fatigue • Body image • Mood • Pain
Mishra et al., 2012	S/M	Cancer patients	4,826	Various exercises	• Health-related QoL • Physical functioning • Role function • Social functioning • Fatigue	

*Note.* I = inhibitors; RCT = randomized controlled trial; S = systematic review; S/M = systematic review including meta-analysis; QoL = quality of life.

#### Exercises

Research showed that supervised exercises and self-managed home-based work-outs are more effective at reducing symptoms of depression compared to the usual care of cancer survivors. While both exercise groups demonstrate a decrease in levels of anxiety, the difference was, unfortunately, not statistically significant due to a small sample size (Levin, Greenwood, Singh, & Newton, 2017). Another study revealed that aerobic exercises improved the quality of life, physical functioning, symptoms of depression and fatigue levels of patients with blood cancer (Bergenthal et al., 2014). Yoga sessions, in comparison to no therapy, are effective at reducing fatigue and sleep issues, along with increasing health-related quality of life among women with breast cancer, although they have no effect on depression and anxiety. When compared to psychosocial/educational interventions, yoga shows statistically significant results of reduced depression, anxiety and fatigue symptom reduction, but no statistical significance was found on health-related quality of life and sleep disturbances (Cramer et al., 2017). Aerobic and resistance exercises have a positive impact on fatigue and cognitive function in women undergoing adjuvant therapy for breast cancer, but have little to no effect on depression (Furmaniak, Menig, & Markes, 2016). The evaluation of dance/movement therapy shows that this sort of therapy is not beneficial for improving depression, anxiety and fatigue symptoms or reducing levels of pain in cancer patients. It increases the quality of life, vigor, and has a positive effect on somatization (Bradt, Shim, & Goodill, 2015). Various types of exercises are associated with improved physical function, role function, social functioning and fatigue. It has also been noted that medium intensity exercises, compared to low intensity exercises, have a greater positive effect on physical functioning, anxiety, fatigue and sleep disturbances (Mishra et al., 2012) (Table 4).

#### Pharmacotherapy

Two randomized controlled trials reported that selective serotonin reuptake inhibitors like escitalopram and citalopram could be effective in preventing major levels of depression in head and neck cancer patients; also, citalopram showed promising results for decreasing levels of suicidality (Lydiatt, Bessette, Schmid, Sayles, & Burke, 2013; Lydiatt et al., 2008). However, one systematic review, including a meta-analysis, failed to find statistically significant favorable effects of antidepressants over placebo (Ostuzzi, Matcham, Dauchy, Barbui, & Hotopf, 2015) (Table 4).

## Discussion

We have established that sociodemographic factors such as: male gender, older age, non-marital status, atheism, lower education, non-employment status and white race are the suicide risk factors for cancer patients. The most important factors for clinical suicide risk are the amount of time after the diagnosis (especially the first year after diagnosis), the localization of the cancer (lung and bronchus, stomach, esophageal, pancreatic, colorectal, oral cavity and pharynx, larynx, head and neck, hematopoietic, genital organs, nasal cavity and sinuses, bone and articular cartilages) and the stage the cancer is in (advanced cancer).

Our research showed that physical and psychosocial suicide risk factors that could be reduced by interventions are pain, poor physical functioning, psychological comorbidity and poor psychosocial support. Physical and psychosocial risk factors are particularly important in developing suicide prevention strategies for cancer patients. Unfortunately, almost all authors of the above mentioned interventional studies reported that the aforementioned studies should be treated with caution due to a lack of high quality research.

Only two of our mentioned studies measured the effect on suicidal thoughts and found that pharmacotherapy (citalopram hydrobromide) and behavioral activation and problem-solving therapy are effective in reducing suicidal ideation; also both have a positive effect on depression. It’s important to notice that the citaloprams’ study was small, only 23 from 36 patients completed the study. Nevertheless, our experience confirms the review data (Li, Fitzgerald, & Rodin, 2012) that anti-depressant drugs together with psychosocial interventions are very effective for severe cases of depression, whereas for cancer patients with mild depression, psychosocial interventions and psycho-education are most useful and could have a positive effect on SRF.

Our study revealed that most exercises are effective in improving one of the SRF – physical functioning. Moreover, supervised exercises, home-based exercises and aerobic exercises have a positive impact on depression and quality of life, while dance/movement therapy didn’t show a positive impact on depression and other mental health qualities like stress, mood and anxiety. These results of the study on dance/movement therapy for cancer patients should be interpreted with caution because the evidence is very low due to the small number of studies included; also the sample size was too small, and one of the studies had a high risk of bias. We found evidence that dance could be effective for emotional and social well-being in a cancer patients group (Sturm, Baak, Storek, Traore, & Thuss-Patience, 2014).

Music therapy, nursing interventions for symptoms control, and yoga are effective in reducing both depression and anxiety so these interventions are very promising at reducing suicide risk. Music intervention, supportive-expressive group therapy, reflexology and telephone-based sessions for care-givers are effective for pain management. Interventions for care-givers have a positive effect on patients’ well-being. One research affirms the importance of care-givers and patients relationship; the study shows that severe depression symptoms correlates with negative family interaction (Oh, Ell, & Subica, 2014).

Telephone-based mindfulness intervention didn’t show significant reduction in psychological distress in men with advanced prostate cancer. However, there are some studies which show that mindfulness-based interventions improve anxiety, depression, stress, sexual difficulties, psychological arousal and immune function in cancer patients (Shennan, Payne, & Fenlon, 2011). For reducing SRF in cancer patients, some other therapies could be effective; for example, there is evidence that art therapy can reduce distress (Lefevre, Ledoux, & Filbet, 2016) anxiety, and depression (Lee et al., 2017). Also there is existing literature that shows hypnotherapy in cancer patients can reduce anxiety, improving sleep and the severity of psychological and physical symptoms for cancer patients (Plaskota et al., 2012).

There are several high quality studies which examine suicide prevention strategies in the general population. In the future, it would be advisable to investigate their effectiveness in the oncological population, for example, investigating the lethal means restriction for cancer patients study (Zalsman et al., 2016). It would also be important to study which interventions could be useful in diminishing demoralization as a risk factor for suicide in cancer patients.

The limitation of this study is that much of the interventional research investigated patients with a specific site of cancer, so it would be difficult to apply the results to all of the oncological population. Another limitation is that there is a lack of research on the direct impact of various interventions on suicidal thoughts and/or behavior. Overall, the most promising interventions for suicide risk reduction in cancer patients appear to be psychotherapy (behavioral activation and problem-solving therapy), nursing interventions, exercises, yoga, music therapy, and interventions by care-givers. For more severe cases, psychosocial interventions together with anti-depressant drugs (citalopram or escitalopram) could be the most promising in reducing suicide risk. Further high quality research is needed to clarify which interventions should be applied and to develop effective suicide prevention strategies for oncology patients.
